# Association of dietary factors with noise-induced hearing loss in Korean population: A 3-year national cohort study

**DOI:** 10.1371/journal.pone.0279884

**Published:** 2022-12-30

**Authors:** Hyun Jin Lee, Juhyung Lee, Chulyoung Yoon, Yesai Park, Young-Hoon Joo, Jun-Ook Park, Young Joon Seo, Kyoung Ho Park

**Affiliations:** 1 Department of Otorhinolaryngology-Head and Neck Surgery, Incheon St. Mary’s Hospital, College of Medicine, The Catholic University of Korea, Seoul, Republic of Korea; 2 Department of biostatistics, Yonsei University Wonju College of Medicine, Wonju, Korea; 3 Department of Otolaryngology-Head and Neck Surgery, College of Medicine, The Catholic University of Korea, Seoul, Korea; 4 Department of Otolaryngology-Head and Neck Surgery, Eunpyeong St. Mary’s Hospital, College of Medicine, The Catholic University of Korea, Seoul, Republic of Korea; 5 Department of Otorhinolaryngology, Yonsei University Wonju College of Medicine, Wonju, South Korea; School of Pharmacy, Ardabil University of Medical Sciences, ISLAMIC REPUBLIC OF IRAN

## Abstract

Noise-induced hearing loss (NIHL) is a hearing impairment (HI) caused by various clinical factors. Identifying the relationship between NIHL and nutrient consumption could help in reducing the prevalence of hearing loss. The aim of this study was to analyze the relationship between NIHL and dietary factors using data of the Korea National Health and Nutrition Examination survey (KNHANES). The data were collected from The Fifth KNHANES 2010–2012. The survey was taken by a total of 10,850 participants aged 20–65 years. Air conduction audiometry was measured at 500, 1000, 2000, and 4000 Hz in both ears. Metabolic syndrome, noise exposure, alcohol consumption, smoking, income level, marital status, and nutritional intake were evaluated. The differences between non-HI and HI participants in the noise-exposed group showed statistically significant differences in age, sex, marital and smoking status, alcohol consumption, and fasting glucose and triglyceride levels (p<0.05). In a multiple regression analysis of the noise-exposed group, age showed a significant association with HI (OR: 0.604; 95% CI: 0.538–0.678) after adjusting for confounders. In multivariate analysis for dietary factors affecting HI in noise-exposed groups, retinol (OR: 1.356; 95% CI: 1.068–1.722), niacin (OR: 1.5; 95% CI: 1.022–2.201), and carbohydrates (OR: 0.692; 95% CI: 0.486–0.985) showed a significant association with NIHL. Age was identified as the only factor significantly affecting NIHL. When the dietary factors of the noise-exposed group were analyzed, high intake of niacin and retinol and low intake of carbohydrates appeared to reduce the risk of hearing loss.

## Introduction

Noise-induced hearing loss (NIHL) is a hearing impairment (HI) caused by continuous noise exposure in the workplace and urban settings. Based on a previous report, approximately 16% of the adult population in Korea is affected by progressive hearing loss (HL) caused by noise exposure [1]. The prevalence of occupational noise exposure was 25% in the U.S. and 20.7% in Korea [2, 3]. NIHL is widely caused by mechanical and metabolic injuries. Major mechanisms of HL include exposure of the cochlea to loud sound causing disruption of the stereocilia on hair cells and cochlear ischemia followed by reperfusion injury, accumulation of reactive oxygen species enhanced by oxidative stress, and excitotoxicity to auditory neuron induced by excessive release of the cochlear afferent neurotransmitter, glutamate. Both mechanisms could slow down the cochlear blood flow and induce apoptosis and necrosis of Corti cells [4].

Many reports have been conducted on the relationship between NIHL and various clinical factors with population-based studies [5]. Agrawal et al. suggested that the NIHL (>25dB, 0.5−4kHz) is associated with age, sex, low education level, cardiovascular disease, and smoking in an analysis of data from the United States Health and Nutrition Survey conducted from 1999 to 2002 (n = 5742) [6]. Engdahl et al. reported a moderate relationship between occupation, HL, and old age in men, based on data from a general adult population sample in Norway (n = 49,948) [7]. Fransen et al. evaluated HL with several clinical factors in a European cross-sectional multicenter study (n = 4083). The noise exposure with occupation and shooting were associated with HL [8].

The clinical factors affecting NIHL include age, sex, smoking, alcohol, and metabolic syndrome (MetS). Among males who work in the cement industry, Somma et al. reported HL of 20 dB in older workers and 5 dB in young workers (n = 184) [9]. Another study showed that higher age and duration of occupational noise exposure are associated with severe HL [10, 11]. A previous study suggested that smoking can accelerate NIHL; the non-smoker group showed better hearing threshold at 4000 Hz than did the smoker group [12]. Nomura et al. reported the effects of smoking on NIHL in a cross-sectional study conducted among Japanese males (n = 397) with occupational noise exposure at a metal factory. The odds ratios (OR) of HL were 3.16 for past smokers and 3.39 for heavy smokers compared with non-smokers [13]. A previous cross-sectional study also suggested that long-term exposure to noise is associated with alcohol consumption and smoking [14]. There have been several reports that suggest that male sex affects NIHL [15, 16]. Helfer suggested that men showed more HL than women after noise exposure to over 85 dB among 804,535 soldiers [17]. MetS include abdominal obesity, high blood pressure, elevated fasting glucose levels, and dyslipidemia [18]. Kim et al. reported that MetS was associated with high-frequency HL in the noise-exposed group [19].

Previous studies have demonstrated the protective effects of consumption of several nutrients such as β-carotene, folate, magnesium, and vitamin C on HL [20, 21]. Free radicals can cause inner ear hair cell damage, while antioxidants have been reported to have an anti-inflammatory effect [22]. Similarly, Kopke et al. found that antioxidants help in reducing hearing damage after exposure to intense noises [23]. Studies have also suggested the association of vitamins B9 (folate) and B12 with HL [24]. A healthy diet rich in many nutrients may provide protection against HL through several mechanisms, including regulation of oxidative stress, protection of cochlear blood flow, reduction of neuroinflammation, and neurodegeneration of auditory nerve fibers and central auditory pathways [25].

Although previous research has shown the association of NIHL with various risk factors, its association with dietary factors has not been investigated in population-based studies. Identifying the relationship between NIHL and dietary factors could help reduce the prevalence of HL. Thus, the objective of the present study was to evaluate the demographic and dietary factors responsible for HL among the noise-exposed South Korean populations, based on the Korea National Health and Nutrition Examination survey (KNHANES).

## Methods

### Study population

**Fig 1:**
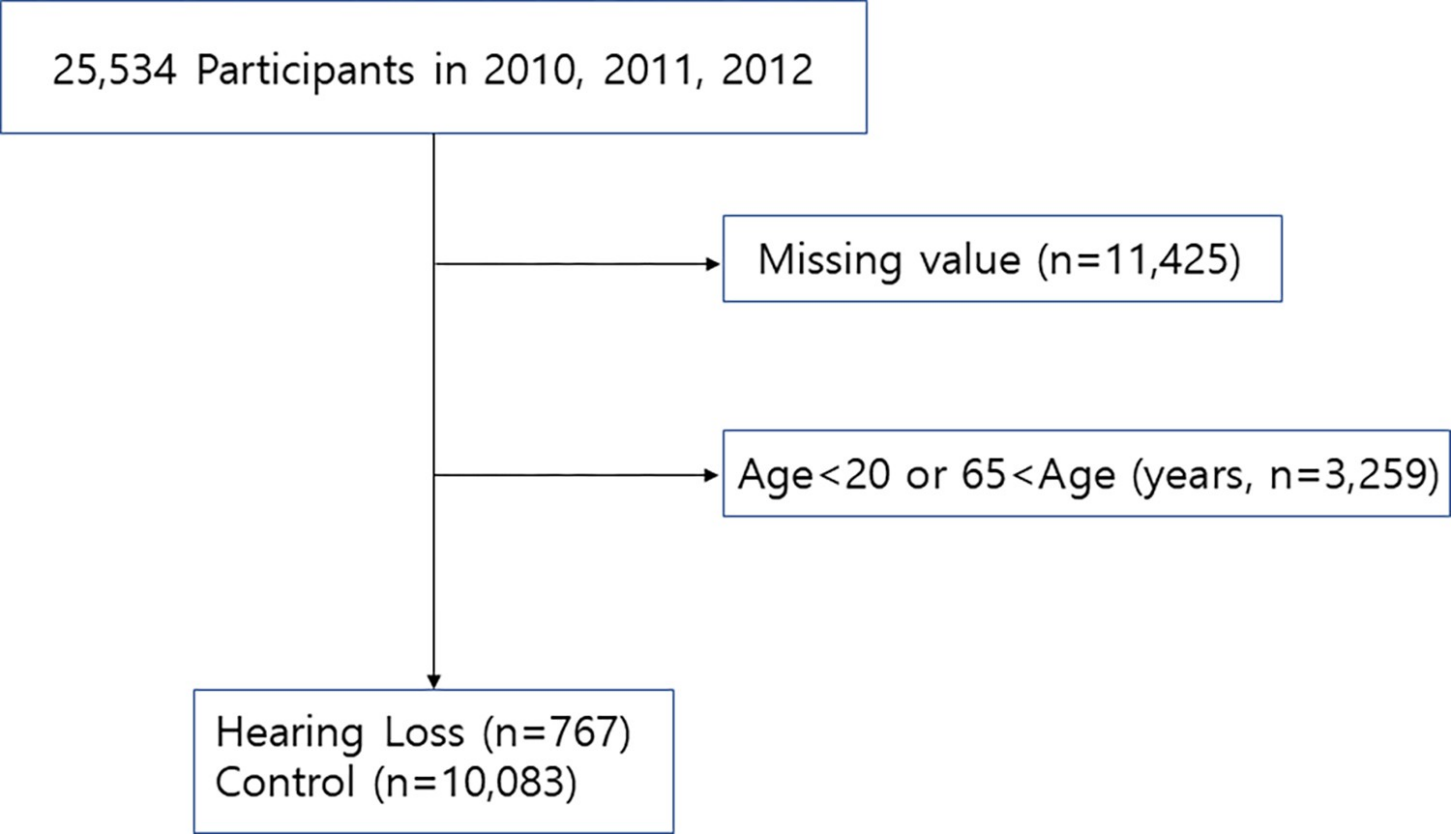
A schematic illustration of participant selection in the present study.

Data were collected from The Fifth Korea National Health and Nutrition Examination survey (KNHANES V) 2010–2012. The survey was taken by a total of 25,524 participants, aged 20–65 years. A total of 11,425 participants with missing data for auditory assessment were excluded. The final study population consisted of 10,850 subjects; 767 subjects with HL and 10,083 subjects without HL (Fig 1). We compared the demographic and clinical characteristics of all subjects (n = 10,850) with and without HI. Furthermore, we conducted an additional analysis by selection of only noise-exposed subjects (n = 1,315) among all participants.

All procedures contributing to this study complied with the ethical standards of the relevant national and institutional committees on human experimentation and with the 1975 Declaration of Helsinki, as revised in 2008. All procedures were approved by the Korea Centers for Disease Control and Prevention Institutional Review Board, and all participants signed a written informed consent form.

KNHANES, a cross-sectional survey, was conducted on a nationally representative sample of the non-institutionalized civilian population in South Korea. This population-based survey included three assessments: health interview and examination and nutrition survey, for a complex, multi-stage sampling design. The survey has contributed to the development and evaluation of health policies and programs, facilitated the establishment of reference values, such as growth charts and dietary references, and further supported health research for the Korean population [26]. The study was approved by the Institutional Review Board of the CMC Clinical Research Coordinating Center (IRB approval number: KC22ZADI0486).

### Auditory evaluation

Audiometry was performed by using SA 203 audiometer (Entomed; Malmö, Sweden). Air conduction audiometry was measured at 500, 1000, 2000, and 4000 Hz in both ears. Testing was carried out in a test booth, in accordance with the requirements of AS/ NZS 1269.4:1998. Pure-tone average was defined as the mean value of measurements taken at 500, 1000, 2000, and 4000 Hz frequencies. Hearing threshold at more than 40 dB in both ears was regarded as symmetric HL because adults with a permanent unaided hearing impairment above 40 dB in the better ear are considered to have a disabling hearing impairment [27, 28]. Since age-related hearing loss could be a confounding factor, those over 65 years were excluded from this study [29, 30].

### Survey for metabolic syndrome

We used the definition of Metabolic syndrome(MetS) from the revised National Cholesterol Education Program’s Adult Treatment Panel III (NCEP ATP III) proposed by the American Heart Association /National Heart, Lung, and Blood Institute (AHA/NHLB) for subjects who meet at least three of the following criteria: (1) abdominal obesity (waist circumference > 90 cm for men; > 80 cm for women), according to the International Obesity Task Force criteria for the Asia–Pacific population (ref), (2) triglycerides (TG) ≥ 150 mg/dL, (3) HDL cholesterol (HDL-C) (< 40 mg/dL for men; < 50 mg/dL for women); (4) hypertension (systolic/diastolic blood pressure ≥ 130/85 mmHg, and (5) fasting plasma glucose (FPG) (≥ 100 mg/dL).

### Data collection

Subjects were asked to complete the self-reported questionnaires about exposure to occupational noise, frequencies of alcohol consumption and smoking status, income level, and marital status. This was done to select subjects who had chronic noise exposure, using the following question: “Have you ever worked in a place with loud machinery or generator noise for more than 3 months?” We classified alcohol intake into four categories: (1) No drink (defined as those who never consumed alcohol in their lifetime.), (2) Drinking alcohol 2–4 times a month, (3) Drinking alcohol 2–4 times a week, and (4) Drinking alcohol more than 4 times a week. Smoking was categorized based on lifetime experience: (1) less than five packs, (2) more than five packs, and (3) no smoke. The average household income was calculated based on the median income of the study participants and was classified into three groups (low, middle, high). Lastly, the participants’ marital status was classified into unmarried, married, divorced or separated.

### Nutritional intake data

The nutrition survey of KNHANES was used to assess 24-hour nutritional intake, dietary habits, and food frequency. Analysis of data was done by trained dietitians via a follow-up interview, a week after the health examination. The mean daily intake of energy over three 24-hour recalls was calculated using the Korean Foods and Nutrients Database of the Rural Development Administration. The average nutrient value contained in each food item, nutrient intake per day, and investigated type, amount, and frequency of food consumed during the day of the survey were classified into types of converted nutrients as follows: energy, water, protein, fat, carbohydrates, fiber, ashes, calcium, phosphate, iron, sodium, potassium, carotene, vitamin A, retinol, vitamin B1, riboflavin, niacin, and vitamin C.

### Statistical analysis

In descriptive analysis of survey data, the sample weighted means and percentages with standard errors for estimates are calculated. Summary statistics presented categorical variables as frequencies and percentages. The two-sample t-test and chi-squared test were used to compare groups using Proc Surveyfreq in SAS. For multivariable analysis, a multinomial logistic regression analysis was performed to test the association between HL and risk factors using PROC SURVEYLOGISTIC in SAS. We repeated multiple logistic regression analyses according to the dietary factors for NIHL. A p-value of less than 0.05 was considered statistically significant, and all statistical analyses were conducted using SAS 9.4 version (SAS Inc., Cary, NC, USA).

## Results

Comparisons of demographic and clinical characteristics between subjects with and without HI are depicted in Table 1. Among the age groups ranging from 20 to 64 years, there was a significant difference in the distribution by age group between the two groups (p<0.001). The distribution of sex showed a significant difference associated with non-HI and HI (p<0.001). The male participants had a higher HI prevalence than female participants. The ratio of noise- to non-noise-exposed groups was significantly higher in the HI (p<0.001) group. Among clinical characteristics described in Table 1, income level (p = 0.0479), marital (p = 0.0153) and smoking status (p<0.001), and MetS (p<0.001) were significantly associated with an increase in the prevalence of HI. However, alcohol consumption and high-density lipoprotein (HDL-C) did not show any statistical differences between subjects with and without HI. The non-HI and HI participants in the noise-exposed group showed statistically significant differences in age (p<0.001), sex (p = 0.076), marital (p = 0.0078) and smoking status (p = 0.076), alcohol consumption (p = 0.0104), and fasting glucose (p = 0.0269) and triglyceride levels (p = 0.0447) (Table 2).

**Table 1:** Comparisons of demographic and clinical characteristics between subjects with and without hearing impairment.

	No hearing impairment (n = 10,083)	Hearing impairment (n = 767)	p-value
Age (years, mean±SD)	54.38 ± 9.6	54.63 ± 9.7	<0.0001*
20–24	610(6.05%)	9(1.17%)	
25–29	773(7.67%)	14(1.83%)	
30–34	1055(10.46%)	22(2.87%)	
35–39	1494(14.82%)	22(2.87%)	
40–44	1295(12.84%)	45(5.87%)	
45–49	1107(10.98%)	62(8.08%)	
50–54	1304(12.93%)	119(15.51%)	
55–59	1174(11.64%)	167(21.77%)	
60–64	1271(12.61%)	307(40.03%)	
Sex (n, Male: Female)	3,858(38.2%): 6,225(61.8%)	385(50.1%): 382(49.9%)	<0.0001*
Noise exposure (n, Yes: No)	1,179(11.6%): 8,904(88.4%)	136(17.7%): 631(82.3%)	<0.0001*
Income (n)			0.0479
Low	3946(39.14%)	322(41.98%)	
Middle	2029(20.12%)	167(21.77%)	
High	4108(40.74%)	278(36.25%)	
Marriage (n)			0.0153
Unmarried	7905(78.4%)	653(85.14%)	
Married	1070(10.61%)	22(2.87%)	
Divorced/Separated	1107(10.98%)	92(11.99%)	
Smoking (n)			<0.0001*
No smoker	6298(62.46%)	404(52.67%)	
Less than 5 packs	278(2.76%)	13(1.69%)	
Over 5 packs	3503(34.74%)	349(45.5%)	
Unknown	4(0.04%)	1(0.13%)	
Alcohol (n)			0.9007
No drink	2287(22.68%)	228(29.73%)	
2–4 times / month	5730(56.83%)	358(46.68%)	
2–4 times / week	1440(14.28%)	106(13.82%)	
Over 4 times / week	572(5.67%)	71(9.26%)	
Unknown	54(0.54%)	4(0.52%)	
Metabolic syndrome (n, Yes: No)	1,854: 8,229	201: 566	<0.0001*
Waist (n, Yes: No)	3,266: 6,817	294: 473	<0.0001*
HDL-C (n, Yes: No)	3,941: 6,142	315: 452	0.3160
Fast glucose (n, Yes: No)	2,287: 7,796	267: 500	<0.0001*
Hypertension (n, Yes: No)	8,849: 1,234	150: 617	<0.0001*
Triglyceride (n, Yes: No)	2,566: 7,517	254: 513	<0.0001*

* Significant at p<0.05, n = number, SD = standard deviation

**Table 2:** Comparisons of demographic and clinical characteristics between subjects with and without hearing impairment in noise-exposed group.

	No hearing impairment (n = 1179)	Hearing impairment (n = 136)	p-value
Age (years, mean±SD)	43.83 ± 12.16	54.41 ± 10.0	<0.0001*
20–24	43(3.65%)	0(0%)	
25–29	81(6.87%)	2(1.47%)	
30–34	117(9.92%)	1(0.74%)	
35–39	141(11.96%)	2(1.47%)	
40–44	151(12.81%)	9(6.62%)	
45–49	143(12.13%)	16(11.76%)	
50–54	192(16.28%)	19(13.97%)	
55–59	155(13.15%)	31(22.79%)	
60–64	156(13.23%)	56(41.18%)	
Sex (n, Male: Female)	720:459	99:37	0.0076
Income (n)			0.4622
Low	520(44.11%)	54(39.71%)	
Middle	261(22.14%)	29(21.32%)	
High	398(33.76%)	53(38.97%)	
Marriage (n)			0.0078
Unmarried	922(78.2%)	119(87.5%)	
Married	119(10.09%)	3(2.21%)	
Divorced/Separated	138(11.7%)	14(10.29%)	
Smoking (n)			0.0282
No smoker	510(43.26%)	43(31.62%)	
Less than 5 packs	30(2.54%)	3(2.21%)	
Over 5 packs	639(54.2%)	90(66.18%)	
Unknown	0(0%)	0(0%)	
Alcohol (n)			0.0104
No drink	212(17.98%)	35(25.74%)	
2–4 times / month	614(52.08%)	65(47.79%)	
2–4 times / week	240(20.36%)	20(14.71%)	
Over 4 times / week	111(9.41%)	14(10.29%)	
Unknown	2(0.17%)	2(1.47%)	
Metabolic syndrome (n, Yes: No)	239:940	34:102	0.1980
Waist (n, Yes: No)	381:798	49:87	0.3820
HDL-C (n, Yes: No)	433:746	55:81	0.3958
Fast glucose (n, Yes: No)	303:876	47:89	0.0269
Hypertension (n, Yes: No)	190:989	26:110	0.3709
Triglyceride (n, Yes: No)	360:819	53:83	0.0447

* Significant at p<0.05, n = number, SD = standard deviation

Table 3 shows the results of multiple logistic regression analyses for HI in the noise-exposed group after adjusting for sex, age, income level, marital and smoking status, alcohol consumption, and MetS. Age showed a significant association with HI (p<0.001); this association indicates that the older participants were at a higher risk of developing HI than did the younger ones (B: -0.5044; OR: 0.604; 95% CI: 0.538–0.678), after adjusting for confounders.

**Table 3:** Multiple regression analysis for variables affecting hearing impairment in noise-exposed group.

	B	OR (95% CI)	p-value
Sex	0.3634	1.438(0.733–2.822)	0.2905
Age	-0.5044	0.604(0.538–0.678)	<0.0001*
Income	-0.1321	0.876(0.706–1.087)	0.23
Marriage	-0.0284	0.972(0.714–1.323)	0.8568
Smoking	-0.2349	0.791(0.569–1.098)	0.1611
Alcohol	0.1033	1.109(0.904–1.36)	0.3208
Metabolic syndrome	-0.0465	0.955(0.617–1.477)	0.8345

* Significant at p<0.001

We performed multiple regression analysis for all dietary factors affecting HI in the noise-exposed group. In Table 4, participants in the noise-exposed group who had consumed higher amounts of niacin (B: 0.4053; OR: 1.5; 95% CI: 1.022–2.201; p<0.001) and retinol (B: 0.3046; OR: 1.356; 95% CI: 1.068–1.722; p<0.001) showed a lower possibility of developing HI, while those with higher consumption of carbohydrates (B: -0.3677; OR: 0.692; 95% CI: 0.486–0.985; p<0.001) were correlated with HI development.

**Table 4:** Multiple regression analysis for all dietary factors affecting hearing impairment in noise-exposed group.

Income	B	OR (95%CI)	p-value
Energy (Kcal)	0.1463	1.158(0.733–1.829)	0.5307
Water (g)	0.0682	1.071(0.809–1.416)	0.6331
Protein (g)	-0.1532	0.858(0.541–1.362)	0.5155
Fat (g)	-0.031	0.969(0.698–1.347)	0.8536
Carbohydrates (g)	-0.3677	0.692(0.486–0.985)	< 0.001*
Fiber (g)	-0.0229	0.977(0.72–1.327)	0.8835
Ashes (g)	-0.0395	0.961(0.626–1.475)	0.8567
Calcium (mg)	0.0557	1.057(0.777–1.439)	0.7232
Phosphate (mg)	-0.0211	0.979(0.604–1.588)	0.9317
Iron (mg)	0.0817	1.085(0.79–1.491)	0.6142
Sodium (mg)	0.0222	1.022(0.738–1.417)	0.8939
Potassium (mg)	0.114	1.121(0.717–1.753)	0.6174
Vitamin A (μgRE)	-0.00192	0.998(0.703–1.417)	0.9914
Carotene (μg)	0.1434	1.154(0.761–1.751)	0.5003
Retinol (μg)	0.3046	1.356(1.068–1.722)	< 0.001*
Vitamin B1 (μg)	-0.1723	0.842(0.608–1.165)	0.2993
Riboflavin (mg)	-0.3164	0.729(0.51–1.041)	0.0817
Niacin (mg)	0.4053	1.5(1.022–2.201)	< 0.001*
Vitamin C (mg)	-0.0307	0.97(0.718–1.309)	0.841

* Significant at p<0.001

## Discussion

Based on the reported epidemiologic and clinical factors, age has been the most significant risk factor for NIHL. In addition, results from the nutrient analysis indicate significant relationship between NIHL progression and consumption of retinol, niacin, and carbohydrate, with NIHL progression showing a negative correlation with retinol and niacin consumption and a positive correlation with carbohydrate consumption.

As a non-modifiable factor, age showed an increase in the risk of developing NIHL. Thus, older subjects are more vulnerable to NIHL than younger ones. A previous study showed that not only an independent but also a causal association between age and factors such as smoking habits, serum cholesterol, systolic or diastolic blood pressure, and analgesic use, is important in the development of NIHL among subjects exposed to hazardous noise levels at work. In our study, among the risk factors associated with NIHL, including age, income, marital and smoking status, alcohol consumption, and MetS, the results demonstrated age as the main risk factor for NIHL prevalence with adjusted confounders. Among noise-exposed workers, Somma et al. reported a 20 dB HL in the older ones, and 5 dB HL in the younger ones, compared to the non-noise-exposed group [9]. Golmohammadi et al. evaluated NIHL prevalence among tractor manufacturing workers (n = 1062) and showed that age and work experience were related to HL [11]. Hederstierna and Rosenhall evaluated an association between NIHL and age in a prospective study of subjects aged 70–75 years. This study suggested that increasing age had more influence on NIHL as an addictive model than noise exposure alone [31]. Furthermore, previous studies found that accumulation of oxidative damage has contributed significantly to the aging process [32].

Oxidative stress, as one of the most extensively studied factors leading to NIHL, occurs because of an imbalance between free radicals and antioxidants in one’s body. To explain its crucial role in NIHL, studies have shown an increase in reactive oxygen species (ROS), reactive nitrogen species (RNS), and lipid peroxides after chronic occupational noise exposure, leading to HL [33]. The imbalance between ROS and RNS is the main element inducing both apoptosis or cell necrosis in NIHL. Moreover, increase in aerobic respiration and utilization of oxygen in mitochondria generates larger amounts of superoxide and other ROS, which have been proven to induce a reduction in the cochlear blood flow [34, 35].

Nutritional diet can play a crucial role in mitigating the effects of NIHL. The role of nutrition in preventing NIHL indicates that increased consumption of antioxidant vitamins could decrease the formation of free radicals, thereby ultimately reducing the prevalence of NIHL owing to its protective and therapeutic effects. Antioxidants, such as vitamins A, C, and E, and the mineral selenium have been proven to protect the body against damage caused by free radicals [4]. Kopke et al. examined the effects of immediate administration of N-acetylcysteine (NAC) and salicylate following noise exposure, and reported a small but significant reduction in NIHL. However, no reduction in sensory cell loss was found [23]. In addition, Yamashita et al. reported the effects of salicylate and vitamin E on preventing NIHL [36]. In a systematic review, treatment with vitamin E and salicylate was shown to be more effective than the treatment with NAC and salicylate in NIHL. Additionally, vitamin B12, folic acid, and NAC may have a protective effect on occupational NIHL [24]. Furthermore, studies have shown that magnesium and other minerals have prophylactic effects in preventing NIHL [37, 38].

Our results showed the effect of vitamin A consumption in lowering the risk of NIHL. The relationship between NIHL and vitamin A has been reported to be inversely proportional to the prevalence of hearing impairment; adjusted OR for highest quartile compared with lowest: 0.51 (CI: 0.26–1.00; p = 0.03) [39]. Furthermore, increased serum levels of retinol and provitamin A carotenoids were clearly associated with a decreased prevalence of hearing impairment [39]. Owing to a high concentration of retinol in the inner ear, retinoic acid, an active metabolite of retinol, contributes to the development of the organ of Corti; thus, it has an anti-apoptotic role in NIHL [39]. Kwak et al. verified the differences among selective agonists of retinoic acid receptors (RARs) in NIHL, in which these agonists demonstrate comparable protective effects against NIHL to retinoic acid [40].

Although a direct association between niacin and NIHL has not yet been studied, previous studies have reported the association between niacin and age-related HL (ARHL). In a rat model of stroke, niacin treatment significantly not only increased the brain-derived neurotrophic factor (BDNF), but also synaptic plasticity and axonal growth [41]. The crucial role of spiral ganglion neurons in endogenous neurotrophic support is further studied with its significant survival rate from BDNF-loaded nanoparticles [42]. Jung et al. stated that the higher intake of niacin is inversely associated with ARHL prevalence in the elderly population (niacin OR, 0.72; 95% CI, 0.54–0.96; p = 0.025, retinol OR 0.66; 95% CI, 0.51–0.86; p = 0.002). The study suggests that the recommended intake levels of niacin and retinol may prevent ARHL in the older population [43]. Higher dietary niacin intake is associated with greater vascular endothelial function related to lower systemic and vascular oxidative stress among healthy middle-aged and older adults [44]. In addition, the interruption of vascular flow of the inner ear in endothelial function is also known to cause idiopathic sudden sensorineural HL [45]. Kaplon. et al. suggested that higher intake of niacin is associated with greater vascular endothelial function and could decrease systemic and vascular oxidative stress [44].

In the present study, the intake of carbohydrates has shown to be inversely associated with NIHL. The relationship between carbohydrates and HL has not yet been fully discovered. However, carbohydrates are known to be contained in unhealthy diets when compared to that present in whole grains, vegetables, and fruits in healthy diets owing to their composition of simple sugars (monosaccharides and disaccharides) with higher levels of triglycerides [46, 47]. A healthy diet could protect the oxidative stress in auditory nerve fiber, neurodegeneration, and neuroinflammation [21]. Excessive consumption of carbohydrates is related with cardiovascular disease, type 2 diabetes, MetS, and obesity [48]. Refined grains contain a considerable amount of carbohydrates, and when consumed in large amounts, the inflammatory response is increased [49].

Moreover, macronutrient intake can aggravate oxidative stress and inflammatory reaction in cell signal pathways [50]. Therefore, carbohydrate diets are linked to the elevation of oxidative stress with chronic inflammatory reactions [51]. A previous study suggested that a significant correlation was noted between high glycemic levels and the presence of HL in a group of adults [52]. Gopinath et al. suggested that higher carbohydrate diet was significantly associated with HL aggravation (P<0.05) [53]. In our study, the association between carbohydrates and noise-induced HL has been revealed, and further studies to explore this association should be conducted.

This study has some limitations. First, it is difficult to determine the exact occupational noise exposure level by self-reported questionnaires because of differences in understanding and interpretation of individuals. Second, there may be a lack of data to analyze nutritional diet from the 24-hour recall method. In future, it is necessary to examine the relationship between an accurately assessed diet and NIHL with the participants present in their workplace so that the level of exposure to noise can be measured accurately. We have found an association between diet and NIHL, but a direct causal relationship has not been found. This is a simple study of the relationship between NIHL and nutrients according to the noise-exposed group without including age-related variables. Hence, we emphasize the importance of established clinical research and animal model studies for evaluating the mechanisms of action of diet in NIHL. In spite of the current lack of an established treatment, our data of the KNHANES successfully translated to find the association between NIHL and dietary factors. Therefore, future studies with precise criteria for noise exposure and similar outcome parameters are required. In conclusion, the prevention of hazardous occupational noise exposure level at work is important with the aging of population. Further, increasing consumption of retinol and niacin-rich food and decreasing consumption of carbohydrates may lower the oxidative stress in the auditory nerve, thereby decreasing the prevalence of NIHL.
