# Correction: Association of dietary factors with noise-induced hearing loss in Korean population: A 3-year national cohort study

**DOI:** 10.1371/journal.pone.0315448

**Published:** 2024-12-05

**Authors:** Hyun Jin Lee, Juhyung Lee, Chulyoung Yoon, Yesai Park, Young-Hoon Joo, Jun-Ook Park, Young Joon Seo, Kyoung Ho Park

The second and third authors, Juhyung Lee and Chulyoung Yoon, as well as the last four authors, Young-Hoon Joo, Jun-Ook Park, Young Joon Seo, and Kyoung Ho Park, should not have been attributed equal contribution to this work.
